# On Some Minor Surgical Operations Performed in or about the Mouth

**Published:** 1891-11

**Authors:** F. Breese


AMERICAN JOURNAL

—OF—

DENTAL SCIENCE.

VOL. XXV. BALTIMORE, NOVEMBER, 1891. NO. 7.

ARTICLE I.

### ON SOME MINOR SURGICAL OPERATIONS PERFORMED IN OR ABOUT THE MOUTH.

BY F. BREESE, L. D. S., ENG.

A paper read before the Students' Society of the Dental Hospital of London.

*Mr. President and Gentlemen:*

The only definition of minor surgical operations which I have been able to find, says that they "comprise the lesser ones, or those of every day occurrence."

Rigidly adhered to, such a definition would exclude those which I propose to notice in my remarks to-night, for it is precisely because they cannot be said to be of every day occurrence—even in the largest practice—that they have been chosen for description.

But, inasmuch as they undoubtedly come within the range of what we all understand by "minor" operations, a preferable definition might be one where they were spoken of as involving in themselves no danger to life, and which cause but slight disturbance to the general health and avocations of the patient.

In the treatment of some conditions met with in the mouth, surgical operations of small magnitude, but not unimportant in their results to patients, have occasionally to be performed by the dentist, and to these my observations will be mainly confined.

One could wish that a communication to a Society like this should be characterized by originality of ideas and expression, but if that be unattainable, either from the nature of the subject or the unfitness of the writer, then it but remains to make what one has to say as useful as possible; and if the end be the suggestion of a treatment in any slight degree more beneficial to the patient than might in some instances be adopted, I can but hope that the absence of the more brilliant quality will be excused for the sake of the lesser one.

With so much of introduction, let me pass to the consideration of our subject; and first a few words on the opening of alveolar abscesses. It is now a generally accepted principle of surgery that an abscess, in whatever part of the body it occurs, should be opened and drained of its contents as soon as it can be safely done, and moreover, that the drainage should be from its most dependent part, or at the point of greatest fluctuation. Now let us suppose that we meet with, and wish to treat, our first case of alveolar abscess deeply situated, for example, in the lower jaw; that the offending tooth is one which we shall attempt to save by treatment; and that the diagnosis usually a simple matter—has been made out; and then dismiss this part of the case from our minds. Meanwhile, we want not only to act up to our principles of surgery, and to relieve our patients, but to prevent the abscess from opening externally which it threatens to do. How are we to accomplish this? If we turn to some of our text-books for information, we shall be probably told that the "abscess must be opened" or the pus evacuated." Excellent advice as far as it goes. Other books more explicit will tell us that we must do it by making an incision down to the pus inside the mouth and between the cheek and jaw. Of the two forms of lancet commonly used for the purpose, the sickle-shaped and the round-edged, the latter is probably the better one to select; and the incision made should be a free one. To prevent a wound of the facial artery, which accident has been known to happen, the incision should be made parallel to the jaw in its length, so that a cut made in the front of the mouth and one made in the molar region would be nearly at right angle to each other in the same plane, though both conforming to the rule; the knife must be kept as close to the bone as possible; its edges must be turned towards the bone and not away from it; and the cut should be made directly downwards. By attending to these points, no serious hæmorrhage will be likely to follow. In getting our drainage, it is to be noted that we have acted directly contrary to the surgical principle enunciated above, as to draining an abscess from its most dependent point, for we have deliberately adopted an uphill method of doing so. The reason is not far to seek. The carrying out of the principle—a thoroughly sound one in its general tendency— in this particular case, would have necessitated an external incision; and anyone who has seen a patient, especially of the feminine sex, marked by the depressed scar cf a healed sinus, will appreciate the reason why the formation of such is prevented by treating the abscess in the way indicated. Palatal abscesses should be opened from behind forwards; the bone hugged as before; and the cut made parallel to the course of the posterior palatine artery. It is generally desirable to take some means to prevent the sides of the incision from uniting before the cure be complete, and this may be done by inserting between them with conveying forceps, or packing in with a blunt probe, a piece of carbolised lint. One long strip should be used; it should be passed right to the floor of the abscess; and the end cut off short as it projects from the incision. No attempt need be made to pack it tightly; and a day is quite long enough to leave it in without changing. The subject of depressed cicatrices has been touched upon, and we may at this point ask with propriety:—can anything be done in the way of improving the personal appearance of a patient unfortunately disfigured by these? That the answer is in the affirmative, appears clear from a reference to Mr. Reeves's work on Orthopœdic Surgery. Writing on this subject, the author first describes a plan of treatment by which all adhesions are first subcutaneously divided, the depressed skin being then everted so that cicatrix remains raised. Two hare-lip pins are then passed at right angles through the base of the cicatrix and retained in position for three days. Mr. Reeves expressed himself as dissatisfied with cases operated on in this way, because "of the subsequent greater or less reappearance of the depression in consequence of absorption or contraction of the new material," and he then proceeds to describe a method adopted by himself, and which, presumably, is more satisfactory. It consists, he says, "in passing a silver wire subcutaneously around the adhesions, making the ends emerge at the same puncture and tightly drawing and twisting the ends together. The adhesions are thus more or less cut through at the time of the operation, and by giving an extra twist occasionally, the remainder of them will give way. The ends are then cut off close to the puncture and buried in it, and the wire left permanently in situ. The new material thrown out as a result of the operation becomes organized around the wire, and the two together prevent the cicatrix relapsing."

These two little operations have been described, although somewhat out of the scope of my paper, because if any considerable improvement in the appearance can really be effected, it is very desirable that the fact should be more generally known than perhaps it is. I would, therefore, particularly ask gentlemen who have had any opportunities of noting the results obtained after these or other similar operations, to give us the benefit of their experience in the discussion to follow.

If gentlemen will now kindly imagine the abscess, which has occupied us in its different phases so far, to be located in the antrum we shall pass by an easy transition from the operations which have been described to the next task we have in hand, and this is to consider the best way of evacuating the pus which has unhappily formed in our patient's antrum. There are at least three different methods of accomplishing this. The antrum may be reached through the vacated socket of a tooth, a perforation may be made through the canine fossa, or the natural opening into the nose may be enlarged. The last way has so many disadvantages that it is only necessary in referring to it, to say that it is now generally abandoned, so that where a selection has to be made it will lie between the first two. If the cause of the suppuration be due to a diseased root, as it probably nearly always is, then the choice of operation is determined for us, for it will only be necessary to enlarge the socket to obtain a good drainage; but where—and no doubt the cases are rare—the teeth are apparently all sound, it is not quite so easy a matter to decide which course to pursue, but, on the contrary, demands some consideration before making a decision, Now of two things, one. We shall have to extract a sound tooth, and proceed as before by enlarging the socket of one of the roots, or we shall have to make our puncture through the canine fossa If the former plan be adopted, a more dependent drainage will undoubtedly be obtained; but, and the "but" is a large one, at the sacrifice of one of the superior molars. This last is so serious a matter that the alternative plan seems a far preferable one to choose. Mr. Christopher Heath—always a great authority on such subjects—shows a decided partiality for the latter method; but as his grounds for doing so are not the same as the reason just given, it may perhaps be allowable to quote his own words. He says: "After considerable experience of both methods, I prefer the puncture above the alveolus, except where a tooth obviously requires extraction; because I find the aperture is less liable to close up than when made through the alveolus, and because food is less likely to find its way into the antrum." It must be admitted that the reasons given, though doubtless weighty ones from the surgeon's point of view, lose some of their force when looked at from the dentist's, for the entrance of food and the closure of the perforation may both be prevented, by the fitting over the aperture of a little appliance, which in its construction makes no great tax upon the mechanical skill of the maker. However, in the case we have imagined, the method to adopt appears plainly to be through the canine fossa. In those cases—also rare—where the teeth are all lost, the perforation may be either done in the same way or from below. Having determined the conditions which would lead us to adopt one or other of these two modes of operating, let us now pass to their actual performance. When the extraction of a tooth is followed by a flow of pus, the perforation of the antrum is already made for us, and subsequent steps simply consist in enlarging the socket till it is at least 1-6th of an inch in diameter. This is done by means of one of the various forms of antrum-perforator, a trocar, or even as was recommended by Sir W. Fergusson, an ordinary carpenter's gimlet, used in the socket with a driving and screwing movement. Where no communication already exists, the instrument may be used in a similar way to make one. The direction of the force to be applied would vary somewhat according to the site of the root through the alveolus, of which the perforation is to be made. If the tooth extracted be a molar it is better to choose the socket of one of the buccal roots—as any attempt to perforate through the socket of a palatine root, as is recommended by Professor Garretson, would probably result in the antrum being missed altogether, and a communication being established with the nasal cavity. The instrument used must at intervals be withdrawn, and a probe passed to see if the antrum be reached. It is not always possible to tell when this latter event has occurred; on the other hand, it sometimes happens, especially if much force has to be used, that the instrument enters so suddenly that there is a danger of it passing right up through the extent of the cavity, and wounding the orbital plate. To prevent the possibility of such an accident, instruments have been devised, provided with moveable collars, which latter should, before beginning the operation, be fixed at such a point that if the instrument should slip, it cannot go far. Its too sudden entrance may also be guarded against by using the fingers themselves as a stop; but it matters not how the danger be overcome, so long as it be not overlooked.

The perforation through the canine fossa is usually done with a small trocar. It should be made about the region of the second bicuspid, and at a point quite above the extremity of the tooth, in direction it should be chiefly inwards, but also somewhat backwards, and upwards. In carrying out this there is again a danger to avoid which Mr. Heath has pointed out. The height of the palate must be observed before commencing proceedings in order to judge what amount of upward direction to give the puncture, otherwise the floor of the antrum may be injured.

Where all the teeth are missing, if the perforation be done from below and through the thickness of the alveolus, it must not be forgotten that the bone will probably be extremely dense, and it is recommended in these cases to make the opening with a "strong, sharp, spear-headed drill in conjunction with the dental engine" in the first instance, and afterwards enlarge with the trocar.

Our patient's antrum may be left to look after itself now for a time while we pass to the consideration of our next case. The dentist will sometimes be consulted for the treatment of solid growths occurring in the mouth, and such a case now claims our attention. On examining the mouth there is to be seen a small tumour springing from between two of the teeth, and separating them slightly from each other. It is of a lobulated appearance, is hard or only slightly elastic to the touch, is but in a trifling degree more vascular than the surrounding tissue, and has none of the attributes looked for in a malignant growth. On enquiry it may be learnt that it has been there for some time—at least many months, possibly a year or two—and has only lately become troublesome, because of its liability to get bitten on at times. Looking at all the facts, no difficulty will be felt in diagnosing the growth as one of simple epulis. It is to be regretted that the term "epulis" has been so loosely employed up to a very recent period, that it has been used to describe almost any kind of tumor of the jaw, from an epithelioma of the gum, to a small polypus of the same tissue. Now, however, it is generally restricted to a tumour having the characteristics just described, which is of slow growth, innocent in nature, and in structure differs but very little from the gum around.

More time has purposely been spent over the diagnosis of this tumour than has been given to the same point in our other cases, because a mistake might easily be made, and with more serious results.

In the treatment of this, as in any solid growth, our object is twofold. The tumour has not only to be removed, but its recurrence if possible prevented. A rule may be laid down which is beyond criticism, that no 'tinkering' should be indulged in, and remembering that a recurrence of our tumour if it take place it may quite possibly be in a sarcomatous or, though rarely, epitheliomatous form, we see what a grave responsibility might be incurred by any want of thoroughness in our treatment of it. Many and various means of getting rid of these growths have been practiced by different operators. Electrolysis, pressure, caustics, the ligature have all had their advocates. The desire to avoid hæmorrhage is probably at the bottom of the employment of any one of them, in preference to the more simple use of the knife; but the tumour we are dealing with is not of so vascular a nature that we need anticipate any serious bleeding from it. Moreover, as the portion of the alveolus beneath the growth has to be removed, a resort to steel instruments sooner or later must happen. Therefore, not only on the score of thoroughness, but on the principal of not making two bites at a cherry, no attempt is going to be made to press, to burn, or tie away this tumour, and reserve the second part of the treatment for another occasion; but the method advocated will be its excision to its seat of attachment, and then the removal of as much of the basal alveolus as may be necessary. The manner of doing so is simple: The tumour may first be isolated by a nearly circular incision made round its pedicle. The growth and all the tissues included in our incision must then be raised from the bone. The part of the alveolar border and its periosteum thus exposed must then be destroyed and for this purpose nothing better could be used than sharp enamel chisels, while for reaching the more inaccessible portions of the septa, which it is well to remove also, the use of fissure and other bars revolving in the dental engine, will no doubt occur to all. If this part of the operation be done thoroughly, the teeth on either side of the growth may be saved, if it be desirable to save them; but in cases where the growth is found to spring from the periodontal membrane of the tooth, the latter must be sacrificed. There can scarcely be any complication of this little operation, except it be that it may be followed by some amount of hæmorrhage, and on this subject something will be said presently. The removal of large-based epulides generally involves a bone section, and as such cases are treated by the surgeon, they need not detain us here.

We may now pass to what has been found to be the most interesting subject of all so far dealt with, and that is the treatment of cysts. Of the two varieties of cyst found in the mouth and having any relation to the teeth, it is intended to speak only of those known as periosteal cysts. The name is an objectionable one, but it has been given because the cyst is formed beneath the periosteum, or more correctly the peri-odontal membrane of the teeth. These cysts may be of almost any size; generally arise in connection with pulpless teeth or roots; should not be difficult to diagnose, and are best treated on certain distinct lines. In the first place the tooth at fault should be extracted, and the extractor will have the satisfaction of knowing that this time at least he has followed the advice so frequently given in our works on Surgery "to remove the cause." The extraction may or may not be followed at once by the escape of the fluid contents of the cyst; but whether or no it is not all. Further measures consist in making an incision into the cyst from the outside, and then cutting out a section or window of the cyst wall. If the bone be much expanded this can easily be done with a pair of short, blunt pointed scissors; if not, something stronger, *e. g.*, cutting forceps might be used, and perhaps a neater way might be found by the use of the trephining bur. The important point about this part of the operation is to make the piece removed large enough. The interior of the cyst wall has next to be dealt with, the object being its destruction. The opening or window already made may be utilized by introducing through it a pair of forceps with serrated edges, as *e. g.*, artery forceps, and with these attempt to seize and remove what is called the lining membrane of the cyst. If this be not done then the structure may be destroyed by wiping it round with fuming nitric acid, or other strong caustic; care, of course, being taken in the application of such powerful agents. Later treatment consists in keeping the cavity sweet by means of syringing with antiseptics, and it may also be packed with carbolised lint in the same way, and for a similar reason as that given in dealing with our case of abscess. The packing also acts as an aid to granulation. The *rationale* of all the steps of our treatment may not be obvious to all, and it may very properly be asked, "Why are you so anxious to destroy the lining of your cyst?" The answer always given is "because the lining consists of epithelium, and until this is destroyed granulation, and consequently obliteration of the cavity, will not take place." Now let us ask ourselves some questions:—Are we sure that this epithelial lining really exists? In Tomes' classical works we are told that the morbid process resulting in the formation of these cysts is probably identical with that resulting in alveolar abscess, but is less acute. If this hypothesis of their origin be the correct one, should we expect to find —and if we do, on what grounds can we explain the occurrence of—an epithelial lining to the cavity? Again, if there it is, would it not be an easy matter to demonstrate under the microscope? Yet, Mr. Eve, in his lecture on "Cystic Tumours of the Jaws," states that he has examined a number of these cysts, and found, not epithelium, but a layer of granulation cells on their inner surfaces. It may be—as this was written some eight years ago—that later researches, with which I am not acquainted, may have cleared the matter up; but if they have not, and there be really no evidence of the existence of this epithelium, then it is high time we ceased from talking of destroying it; and personally, if told later that in this particular I have advocated treatment somewhat empirical in nature I shall not be able to clear myself of the charge. Let us return to the subject of extraction of the offending tooth. Speaking generally, this is to be urged upon, but, at the same time, cases may arise where the sex of the patient and the position of the tooth renders it desirable to save the latter if it be possible. At least it is possible to try by the excision of part only of the root, as some of us must have seen demonstrated at this hospital in the following way:—A small vertical incision down to the bone is made over the extremity of the root of the affected tooth; a plug of cotton wool dipped in the mastic is placed and fixed in the cut thus made. If the patient be left with this in for a few days, on his or her return, the removal of the plug of wool will find the edges of the cut gaping apart, and thus a better exposure of the part to be operated upon obtained, and in addition the operator will not be bothered with the hæmorrhage from the cut. The next proceeding is to chip away the bone covering the root, when it will most likely be discovered that the extremity of the latter, necrosed or partly absorbed, projects into the cyst cavity. By the excision of this part of the root with fine-cutting pliers, and smoothing it off with burs, by filling the root, and by otherwise treating our cyst on the lines mentioned, there may be a chance of saving the tooth.

It seems appropriate to choose as our last subject, that of hæmorrhage in the mouth, and indeed the paper would hardly be complete without some reference being made to it, but it may at once be said that the great demand already made on the patience of members, prevents any attempt to deal exhaustively with it being made.

We are under this disadvantage in our treatment of hæmorrhage of the mouth, that many of the methods of arresting it practiced in other parts of the body, cannot be employed so directly here. This arises from the relatively greater arterial supply, the more or less inaccessible positions of the vessels, their free anastomosis, and also the greater difficulty in dealing with such in a comparatively confined space like the mouth. Hence we find in surgical practice a frequent resort to the actual cautery as a means of overcoming oral bleeding. The operation described to night will probably not have been followed by any abnormal flow of blood, and the little we have had could be treated by much simpler means to begin with. Thus iced water held in the mouth is usually the first thing tried. Not succeeding with this, we might try mixing an astringent, such as alum, with the water. In a surface such as that left after the removal of an epilus, pressure applied digitally might be employed, and that failing, styptics might be used in conjunction with it in this way:—A piece of lint dipped in an alcoholic solution of tannin should be placed over the raw surface, a dry pad of the same material over that, and firm uniform pressure be kept up for some time over all. Failure need not be anticipated in this particular case, but if the bleeding were still uncontrolled the cautery might then be requisitioned. For ordinary cases this would be sufficient, but if not, later treatment would be to deal with the artery supplying the wounded surface. If this were the facial, no difficulty would be encountered; but the inferior dental in its bony canal is not so easily got at. In this latter connection, Mr. Tomes has suggested the advisability of drilling a hole with the engine into the inferior dental canal—of course between the bleeding point and the carotid trunk—and then plugging the hole thus made with a wooden peg. The last resort need only to be mentioned; it is to tie the common carotid artery. Such a step must be rarely necessary, and in the case of a patient of the hæmorrhage diathesis increasing, as it must, the wounded surfaces, of doubtful benefit.

Hæmorrhage from the socket of a tooth, which may be briefly touched upon, is treated largely, but not entirely on local lines. Local measures consist in applying a well-fitting plug to the socket, with or without styptics. In this, as perhaps in most things, the best results are obtained by careful attention to details. Out of the many methods there are of treating this form of bleeding but one will be here described, and that is the way I have invariably adopted in my own experience. Some plugs of cotton wool of different sizes are rolled up tightly, and half of them are dipped in a solution of tannin, or impregnated with the dry powder of tannic acid. The socket is then cleared of any partly-formed clot, and the cavity syringed with hot water. Then momentarily controlling the bleeding, if it be possible, by firm pressure with a pad of wool, the plugs are rapidly passed to the floor of the socket and packed in alternately, that is to say, first a plug of stypticised wool is placed in position, then a dry one, and so on until the cavity be full. The use of tannin is clean and invariably effectual. A point not to be forgotten is to count the plugs, otherwise, when the time comes for removing them, it may not be possible to know that all have been withdrawn, without—what should be unnecessary—probing, and stirring up of the cavity with the risk of restarting the hæmorrhage. The extraction of teeth with abscess cavities at their roots, if one may say so from a small experience, seem particularly liable to be followed by an unusual amount of hæmorrhage.

The difficulty of getting a plug to remain in is much enhanced in those cases where much force has had to be used in the extraction, and as a consequence the gum is somewhat lacerated and pushed away from the bone, and even perhaps the external plate of the alveolus broken away. To cope with these, pressure has to be resorted to. This may be done by compresses of various materials, such as lint, or gutta-percha being bitten on firmly by the opposing teeth, if such be present; and then, as it is said to be not safe to trust to "the patient's volition," the jaws must be bandaged together. It seems preferable, however, to take a little more trouble and make a vulcanite plate to cap the whole socket and adjacent surfaces. This would be more comfortable for the patient, and would probably answer better in exerting a more uniform and immovable pressure, and could be easily constructed in the course of a few hours. There are certain other measures, not local, it is well to advise the patient on. He should avoid the recumbent position, keep quiet and in the cool air, and should go without stimulating food and liquors for the time being. That this advice is necessary, a case in point will show. The patient came back twenty-four hours after an extraction with the news that the socket was still bleeding freely; when asked how she had spent the intervening time, and why she hadn't returned sooner, she stated that, acting on her landlady's advice, she had been sitting by the fire all day, and had kept herself up—as she called it—

on brandy-and-water. One should never speak ill of a legitimate practitioner, but words concerning that landlady's treatment of hæmorrhage could only be those of condemnation.

Gentlemen,—Our paper draws to a conclusion; the writer cannot congratulate himself on having said very much that is new to you, but at least he hopes his cases have been treated on sound principles. He is almost ashamed to have occupied the time of members for so long on subjects in which so many here must be richer in actual experience than he can lay any claim to be. He hopes that those members will see to it that anyone who may have come here to-night in the hope of deriving some benefit shall not go away disappointed. With this hope, which though hackneyed is none the less sincere, and with his warmest thanks for your attention, he may best bring these observations to an end.—*London Dental Record.*

---

ARTICLE II.

